# A Preliminary Assessment of Global CO_2_: Spatial Patterns, Temporal Trends, and Policy Implications

**DOI:** 10.1002/gch2.202300184

**Published:** 2023-11-16

**Authors:** Ahmed M. EI Kenawy, Talal Al‐Awadhi, Meshal Abdullah, Rana Jawarneh, Ammar Abulibdeh

**Affiliations:** ^1^ Instiuto Pirenaico de Ecologia Consejo Superior de Investigaciones Cientificas (CSIC) Campus de Aula Dei, 1005 Zaragoza 50059 Spain; ^2^ Department of Geography Collage of Arts and Social Science Sultan Qaboos University Muscat Oman; ^3^ Applied Geography and GIS Program Department of Humanities College of Arts and Sciences Qatar University P.O. Box: 2713 Doha Qatar

**Keywords:** CO_2_ emission, economic growth, environmental sustainability, GHGs, PCA

## Abstract

This study offers a comprehensive analysis of the distribution, evolution, and driving factors of CO_2_ emissions from 1990 to 2016 at multiple spatial scales. Utilizing 26 indicators encompassing various facets of CO_2_ emissions, it is employed principal component analysis (PCA) and empirical orthogonal functions (EOFs) to identify the dominant characteristics of global CO_2_ emissions. This model retained three core components, accounting for 93% of the global CO_2_ variation, reflecting emission trajectories and associated economic metrics, such as Gross domestic product (GDP). The analysis differentiated the effects of these components based on countries' economic standings. Using a novel aggregated index, significant national contributors to global CO_2_ emissions are pinpointed. Notably, the leading contributors are found among developed nations (e.g., the United States, Canada, Japan), Gulf states (e.g., Saudi Arabia, Qatar), and emerging economies (e.g., China, Brazil, Mexico). Furthermore, these results highlight that shifts in global CO_2_ emissions over the past 30 years are predominantly influenced by factors like industrial emissions and GDP. Results also demonstrate a distinct relationship between a country's CO_2_ emissions and its physical and socioeconomic factors. Specifically, the nation's coastline length, population density in coastal regions, and the diversity of its climatic conditions significantly influence its carbon footprint.

## Graphical abstract

This study offers a comprehensive analysis of the distribution, evolution, and driving factors of CO_2_ emissions from 1990 to 2016 at multiple spatial scales. Utilizing 26 indicators encompassing various facets of CO_2_ emissions, the study employed principal component analysis (PCA) and empirical orthogonal functions (EOFs) to identify the dominant characteristics of global CO_2_ emissions. The model retained three core components, accounting for 93% of the global CO_2_ variation, reflecting emission trajectories and associated economic metrics, such as income level and Gross domestic product (GDP).

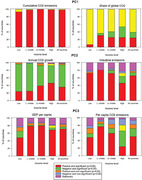

## 1 Introduction

The increasing atmospheric concentration of greenhouse gases (GHGs) is one of the most pressing global problems of the 21st century.^[^ ^1^ ^]^ Most of the increase in GHG emissions can be traced back to human activities, including the burning of fossil fuels for economic growth, industrial emissions, changes in land use, and advances in technology.^[^ ^2^, ^3^, ^4^, ^5^ ^]^ Fossil fuels have been the primary source of anthropogenic emissions to the atmosphere since 1950, and their relative share has increased rapidly and constantly since then.^[^ ^6^ ^]^ For instance, since the onset of the industrial era in 1750, the concentration of carbon dioxide (CO_2_) has surged from approximately 277 parts per million (ppm) to 417.06 ppm in 2022.^[^ ^7^, ^8^, ^9^ ^]^ CO_2_ emissions have more than doubled across the globe from 1990, with an average annual growth rate of 1.8%.^[^ ^10^ ^]^ Following this situation, a worldwide effort is under way to cut GHG emissions. Numerous initiatives and global commitments have been sparked by the United Nations Framework Convention on Climate Change (UNFCCC) to stabilize atmospheric concentrations of greenhouse gases at safe levels.^[^ ^11^, ^12^ ^]^ Reduced Emissions from Deforestation and Degradation (REDD+) was deemed the most important low‐carbon emitting initiative.^[^ ^13^, ^14^ ^]^

CO_2_ emissions are a global problem since countries export their GHG emissions due to the Earth's atmosphere intermixing globally. That creates a problem of inequity between the countries that cause GHG emissions to rise and the countries that suffer the negative effects of those emissions, like climate change. In this context, while developed nations are making advancements in their clean energy as an alternative to fossil‐based energy, less‐developed nations are lagging behind.^[^ ^15^ ^]^ According to Maraseni & Qu,^[^ ^16^ ^]^ the majority (over 50%) of global soil emissions and nearly half (49%) of agricultural‐related emissions are reportedly produced in just seven countries: China, the United States, India, Australia, Brazil, Canada, and Chile. Nonetheless, the impacts of climate change are not uniform across countries, and the effects of GHG emissions will travel far beyond their borders. Depending on how much they contribute to the causes of climate change, some countries that emit lot of GHGs may be more or less vulnerable to its effects. Althor at el.^[^ ^17^ ^]^ investigated the correlation between countries’ GHG emissions and their vulnerability to the negative impacts of climate change, demonstrating a global inequality in this relationship. Specifically, 20 of the 36 highest emitting countries were among the least vulnerable to negative effects of future climate change. However, only 11 of the countries with low or moderate GHG emissions were also vulnerable to the negative effects of climate change, while 28 countries had a good balance between low and high emissions. Further, it is projected that inequality will grow even worse in the future. The ability to ensure sustainable economic growth and wealth while also achieving significantly reduced utilization of energy assets and GHG emissions remains a challenge for every country worldwide.^[^ ^18^ ^]^

Multiple empirical studies have looked at time series data from a variety of countries and regions to draw conclusions about the links between CO_2_ emissions and environmental deterioration, energy use, and other socioeconomic variables.^[^ ^19^, ^20^, ^21^, ^22^, ^23^ ^]^ Examples include Gorus and Aslan^[^ ^24^ ^]^ who used a panel causality test to examine the relationship between CO_2_ emissions, economic growth, and energy use in the Middle East and North Africa (MENA) region from 1975 to 2014. They found a negative long‐run correlation between these variables, stressing that energy protection policies in the region had no negative relationship with economic growth in the intermediate and short‐run. Using panel unit root and panel cointegration, another study by Halicioglu^[^ ^22^ ^]^ looked at the short‐ and long‐run nexus between energy consumption, economic growth, and pollution in six Sub‐Saharan countries of Africa from 1980 to 2014. Also, Dong et al.^[^ ^25^ ^]^ investigated the most influential factors affecting global CO_2_ emissions, demonstrating that renewable energy contributed significantly to global CO_2_ reduction, mainly in South and Central America and Eurasia. Muhammad^[^ ^19^ ^]^ analyzed the dependency between CO_2_ emissions, energy consumption, and economic growth in a sample of developed, Middle Eastern, and emerging countries between 2001 and 2017. They attributed increased economic activity and CO_2_ levels to higher energy consumption in all countries. Exceptionally, the decline in economic growth across the MENA countries coincided with an unprecedented rise in CO_2_ emissions. Also, Kasman et al.^[^ ^26^ ^]^ analyzed the EU's CO_2_ emissions, Gross Domestic Product (GDP) growth, trade openness, energy consumption, and urbanization from 1992 to 2020 and found a causal relationship between all of these factors. According to this study, long‐term CO_2_ emissions were found to be significantly affected by economic growth and trade. An inspection of these studies reveals that they focused mainly on the numerical changes of CO_2_ in high‐emitted countries or compared CO_2_ changes over time between developed and developing countries. Unfortunately, less focus has been placed on initiating spatial and temporal characteristics of CO_2_ emissions using a layered approach (i.e., global vs regional vs national). Utilizing this layered approach facilitates the identification of both overarching patterns and unique anomalies, which is crucial for the development of effective mitigation strategies. Due to the sector‐spanning character of anthropogenic sources of CO_2_ emissions, coordination of mitigation responses has remained a challenge for most of countries worldwide, especially in the developing world. In this respect, the extent to which a given country is able to implement policies to combat climate change depends on country‐specific factors such as its history, institutional framework, and social fabric. This feature makes it important to examine the factors that impact CO_2_ emissions not only on a global scale, but also at regional and national levels.

In the literature, there has been a variety of methods to assess the factors influencing the trajectory of CO_2_ emission, including –for example– the connection between economic growth, information and communication technologies (ICT), and CO_2_ emissions.^[^ ^27^, ^28^, ^29^ ^]^ Many of these studies employed techniques like decomposition analysis, the Kaya identity, and the IPAT identity to isolate the impact of different factors on CO_2_ evolution. A complete review of the studies that fall into this category is included in Taka et al.^[^ ^30^ ^]^ In this study, we applied an opposite procedure to deduct the information on CO_2_ emissions from a wide variety of key determinants and define a smaller number of significant components that summarize all data inputs. Multivariate statistics like the Principal Components Analysis (PCA) can be employed to accomplish this task. It is noteworthy to indicate that the primary aim of employing PCA in our study was not solely to provide a summary of CO_2_ emissions at different spatial scales (global vs regional vs national). Instead, our intention was to reveal underlying patterns and relationships among the different indicators across these various spatial scales. PCA was employed to discern the sources or factors that exhibit a tendency to co‐vary, thereby offering valuable insights into the fundamental mechanisms or common drivers of emissions. Through the examination of extensive patterns in CO_2_ emissions, we can obtain a comprehensive understanding of prevalent trends, predominant sources of emissions, and overall characteristics of various regions or sectors. This comprehension is fundamental to the implementation of any mitigation efforts. In mitigation strategies, a comprehensive comprehension of both macro and micro trends enables policymakers and stakeholders to effectively allocate resources. As such, recognizing and addressing global, regional, and national anomalies ensures that policies and strategies are equitable and do not disproportionately burden or overlook any particular group. Also, while the identification of overarching patterns can be beneficial in guiding extensive endeavors and the allocation of resources, anomalies can serve as indicators for specific areas that require focused investments. Exclusively prioritizing overarching patterns may result in the formulation of generalized policies that could potentially be inequitable for specific regions or sectors that deviate from the prevailing trend.

Overall, this study aims to examine the spatial and temporal distribution of CO_2_ on national, regional, and global scales from 1990 to 2016. Specifically, we aimed to develop an extensive, long‐term inventory of CO_2_ emissions based on 26 indicators at a global scale. Using the PCA and trend analysis, our study delves into the spatial and temporal variations of these global CO_2_ indicators across global, regional, and national scales over the past three decades. Also, we employed the World Bank's classification of countries to analyze CO_2_ emissions, allocating nations into distinct brackets based on their income level, namely high‐income, upper‐middle‐income, lower‐middle‐income, or low‐income categories. This categorization enables a more comprehensive understanding of the distinct attributes and fundamental determinants of carbon dioxide emissions within diverse economic settings. In light of these objectives, our research endeavor seeks to address pivotal questions, namely:

‐ What are the dominant characteristics of CO_2_ emissions at national, regional, and global scales between 1990 and 2016?
‐ Which CO_2_ indicators have been paramount in influencing global, regional, and national emission trends in the past three decades?
‐ How do the identified core components of CO_2_ emissions correlate with economic metrics, such as income level and GDP, as well as geographical settings (e.g., location, climate conditions)?
‐ How can the insights derived from this research be leveraged to inform and optimize future CO_2_ mitigation strategies?

## 2 Expeimental Section

### 2.1 Dataset Description

The empirical data used in this study was retrieved from our world in data repository (https://www.ourworldindata.org/co2‐and‐other‐greenhouse‐gas‐emissions). It is a credible source of data on CO_2_ emissions, as it aggregates information from official international (e.g., the United Nations Statistical Office), national statistical publications, and annual questionnaires, providing up‐to‐date information and more complete picture of energy consumption and CO_2_ behavior globally and at the national level. As such, data from this repository has been widely used in many investigations. Our World in Data is publicly available and contains a wealth of credible data on CO_2_ emissions at both the global and national levels. This database compiles extensive information at the national level, such as CO_2_ emissions, combined with other macroeconomic data like population, population density, GDP per capita, and other similar metrics. Overall, these indicators span a wide range of metrics like annual CO_2_ emissions, shared CO_2_ from different energy sources (e.g., coal, oil, gas, electricity), and economic sectors (e.g., forestry, agriculture, manufacturing, industry, transport, etc.). Also, the level of economic development is represented by GDP per capita, while CO_2_ emission per capita is a measure of environmental impact. The incorporation of multiple environmental and socioeconomic indicators offers a more holistic perspective on the determinants impacting emissions. Herein, it may be argued that the quantification of CO_2_ emissions can be consolidated as an aggregate value derived from diverse origins (e.g., domestic, agricultural, industrial, etc.). However, it is important to note that the specific sources of these emissions and their respective contributions could exhibit substantial variations in both geographical locations and temporal periods. As such, our 26 indicators could encompass the intricacies associated with emissions drivers, taking into account various factors such as economic activities, technological adoption, policies, and other relevant influences.

Also, it should be noted that albeit with the availability of different data sources, e.g. those of the World Resources Institute (WRI), the International Energy Agency (IEA), the US Energy Information Administration (EIA), the Carbon Dioxide Information Analysis Center (CDIAC), and the National Development and Reform Commission (INCCCC and SNCCCC),^[^ ^31^, ^32^, ^33^ ^]^ the preference was made to employ data from only a single source (Our World in data). This was simply because relying on different non‐standard data sources makes it difficult to directly compare the results. The Our World in data dataset was based on comprehensive national vulnerability assessments and comprehensive CO_2_ emissions data.

A list of the 26 indicators employed in this study and their definitions is given in **Table** **1**. The selected indicators were provided at a country level from 1990 to 2016. However, due to missing or inadequate data, it is only included countries with complete data, which resulted in a total of at least 177 countries for each indicator. Herein, it should be emphasized that the exclusion of certain countries with incomplete data from analysis had minimal impact on the findings of this study recalling that most of the eliminated countries corresponded to the world's island and archipelagic nations.

**Table 1:** List of the 26 indicators used in this study and their definitions.

Indicator	Description
Agricultural emissions	The GHGs are released into the atmosphere as a result of human activity in agriculture, including farming and livestock production.
Annual CO_2_ emissions	The amount of CO_2_ that is released into the atmosphere in a given year from human activities such as the burning of fossil fuels, deforestation, and industrial processes.
Annual CO_2_ growth	The increase in the amount of CO_2_ that is released into the atmosphere on a year‐over‐year basis.
Building emissions	The amount of greenhouse gases that are released into the atmosphere from the construction, operation, and maintenance of buildings.
CO_2_ emission flaring	The burning of excess or waste CO_2_ that is produced during industrial processes, such as oil and gas production.
CO_2_ emissions from coal	The amount of CO_2_ that is released into the atmosphere as a result of burning coal for energy.
CO_2_ emissions from oil	The amount of CO_2_ released into the atmosphere as a result of burning fossil fuels, specifically oil.
CO_2_ emissions per unit of energy	The amount of CO_2_ that is emitted for each unit of energy produced or consumed.
CO_2_ emissions from cement	The greenhouse gases produced during the manufacturing of cement, a primary ingredient in concrete.
CO_2_ emissions from natural gas	The CO_2_ carbon dioxide that is released into the atmosphere when natural gas is burned for energy.
CO_2_ emissions from industry	The CO_2_ that is released into the atmosphere as a result of industrial activities.
Cumulative CO_2_ emissions	The total amount of CO_2_ that has been released into the atmosphere as a result of human activities.
Electricity emissions	The CO_2_ and other greenhouse gases are emitted as a result of generating electricity.
Energy consumption per GDP	Also known as energy intensity, is a measure of the amount of energy used to produce one unit of GDP.
Forestry emissions	The GHGs are released as a result of human activities in forested areas, such as deforestation, forest degradation, and the burning of wood for fuel.
Fugitive emissions	Unintentional releases of gases or particulate matter into the atmosphere from sources that are difficult to control or contain.
GDP per capita	Is a measure of a country's economic output that accounts for its number of residents.
Industrial emissions	The release of pollutants and greenhouse gases into the atmosphere from various industrial processes and activities.
Manufacturing emissions	The release of pollutants and greenhouse gases into the atmosphere from the manufacturing process.
Per‐capita CO_2_ emissions	The amount of CO_2_ that is emitted into the atmosphere by an individual person or a population per unit of time, usually measured in metric tons per year.
Per‐capita consumption	The average amount of a particular good or service that is consumed by an individual person in a specific country or region.
Share of global CO_2_ emissions	The percentage of total global CO_2_ emissions that are produced by a particular country, region, or group of countries.
Share of global cumulative CO_2_ emissions	The percentage of total global CO_2_ emissions that have been produced by a particular country, region, or group of countries over a specific period of time.
Transport emissions	The release of pollutants and greenhouse gases into the atmosphere from the transportation sector, including cars, trucks, buses, trains, ships, and airplanes.
Waste emissions	The release of pollutants and greenhouse gases into the atmosphere from the disposal or treatment of waste materials.
Other fuel emissions	The release of pollutants and greenhouse gases into the atmosphere from the use of non‐fossil fuel sources.

From a temporal perspective, while data on CO_2_ emissions traced back to the early decades of the 20^th^ century, we restricted our analysis to data spanning the period between 1990 and 2016. Our decision was made to employ data for the last three decades to minimize uncertainties introduced in the early‐decades data, which may have originated from reconstruction procedures. Moreover, a more accurate estimation of CO_2_ emissions has been possible in recent decades, mainly due to advancements in CO_2_ estimates. Unfortunately, we restricted our analysis to the year 2016 since a comprehensive new data set for the most recent years was not readily available. Herein, it is noteworthy to indicate that we applied a standardization procedure to all CO_2_ indicators. This method is critical for reducing the impact of the varying data ranges for specific indicators, especially between the most and least developed countries, and for resolving the problem of inconsistent unit usage across the different metrics. Also, this procedure allowed for better comparability, classification, and quantification of the different metrics. Herein, the data were simply standardized by considering the mean and standard deviation of all values for each independent indicator, as follows:

(1) $$Z=\frac{x-\mu }{\sigma }$$

where

(2) $$\mu =\frac{1}{N}\sum_{i=1}^{n}\left(x_{i}\right)$$

and

(3) $$\sigma =\sqrt{\frac{1}{N}\sum_{i=1}^{N}{\left(x_{i}-\mu \right)}^{2}}$$

where Z refers to the standardized unit (z‐score), *x* is the value of the indicator in the year *i*, μ is the mean, σ is the standard deviation.

### 2.2 Spatial Patterns of CO_2_ Emissions

Utilizing a comprehensive compilation of 26 indicators pertaining to CO_2_ emissions, PCA proved to be an effective method for the consolidation of the majority of the variability present in our dataset by reducing the number of dimensions and thereby promoting a more straightforward process of interpretation and analysis. Furthermore, this procedure was important to account for the high colinearity that may be presented between the different indicators. Herein, it is employed a principal components analysis (PCA) in an S‐mode to determine the most dominant spatial patterns of CO_2_ indicators on the global scale. Simply, it is considered the mean, standard deviation, and coefficient of variance for each country and individual indicator over the study period as inputs for the PCA. With the help of the empirical orthogonal functions (EOFs), only the most significant components (i.e., those with eigenvalues greater than 1) were kept in the final model. The orthogonality of PCA ensures that each principal component represents a unique variance in the data. This characteristic makes it easier to discern the distinct influences of each component, reducing the risk of multicollinearity. In this work, the Varimax technique was used to rotate the remaining data components in order to reduce the data dimensions and capture the maximum variation. Then, for each retained (significant) component, the best‐correlated indicators was kept with this component and mapped the scores corresponding to all countries within this component.

In order to give an overall picture of the contribution of each country to global CO_2_ emissions from different perspectives (i.e., environmental, economic, demographic, etc.), it is introduced a novel composite CO_2_ indicator that accounts for the weight of each country in every specific indicator. Herein, our developed indicator accounts for the rank of each country on the scale (0‐1) corresponding to each individual indicator rather than their absolute values of this indicator. Specifically, this composite indicator (CI) was computed as:

(4) $$CI=\mathrm{mean}\left(SI_{1},SI_{2},SI_{3},SI_{4},\;\text{…},\;SI_{n}\right)$$

where

(5) $$SI\;=\frac{{\mathrm{seq}}_{i}-1}{c-1}$$

where *SI*_1_ is the score for indicator 1, *n* is the total number of indicators employed in this study (*n = 26*), *c* is the total number of countries per an individual indicator (*c = 177*) and *seq* is the sequence number of each country within this indicator.

Following this procedure, for each single indicator, a country is given a score between 0 and 1, where 0 denotes the country with the least values on this indicator, while 1 refers to the countries with the highest values. Finally, taking into account the scores of all indicators, it is calculated an aggregated indicator (AI), which summarizes the overall picture of CO_2_ emission metrics in each country, with respect to all countries worldwide. The AI was computed through a simple averaging of the 26 CIs. Values for this aggregated index range from 0 (the least contributor) to 1 (the most contributor to CO_2_ emissions). To compute this aggregated indicator, an equal weight was given to all (*N* = 26) CIs. As the determinants to CO_2_ emission growth could vary from one country to another, this simple average can minimize the possible effect of the different weights of indicators, due to differences between individual countries' mitigation policies, on the calculation of the aggregated index.

### 2.3 Trend Analysis of CO_2_ Emission Indicators

The amount of change in the different CO_2_ indicators was calculated for the period 1990–2016 using the linear least squares regression technique. For each investigated indicator and country, the slope was used to quantify change per unit of time, with larger slopes indicating more change and vice versa. To determine whether or not the observed changes were statistically significant at the 95% confidence interval (*p* < 0.05), the modified Mann–Kendall statistic was employed.^[^ ^34^ ^]^ This non‐parametric test does not assume any prior data distribution. The modified Mann–Kendall test is preferable to the traditional Mann–Kendall test because it limits the possible impact of serial autocorrelation introduced in the data on trend assessment. This occurs because this test applies a correction factor to the original variance formulation, taking into account the sample size.

To eliminate the influence of statistically significant autocorrelation coefficients, a modified variance of the Mann–Kendall test (S), designated as Var (S)^*^ is computed as:

(6) $$Var\;{\left(S\right)}^{*}=\;V\;\left(S\right)\frac{n}{n^{*}}$$

where n* is the effective sample size and the n/n* ratio was computed following the formula given by,^[^ ^34^ ^]^ as follows:

(7) $$\frac{n}{n^{*}}=\;1+\frac{2}{n\;\left(n-1\right)\left(n-2\right)}\;\sum_{i=1}^{n-1}\left(n-1\right)\left(n-i-1\right)\left(n-i-2\right)ri$$

where *n* is the actual number of observations, ri = lag‐i significant autocorrelation coefficient of rank i of time series. The Mann‐Kendall Z was then used to determine whether or not the trend was statistically significant at the 95% confidence level.

Finally, to facilitate comparison between CO_2_ emissions indicators on a global scale, the World Bank classification of countries was adopted. The World Bank has made this classification based on the average annual national income, categorizing nations into distinct income brackets, including low‐income, lower‐middle‐income, upper‐middle‐income, and high‐income. Further details about this classification can be found via https://www.blogs.worldbank.org/opendata/new‐world‐bank‐country‐classifications‐income‐level‐2022‐2023. This choice to implement this classification system was motivated by extensive factors. First, this classification provides a systematic and internationally‐accepted approach for categorizing countries according to their economic status. Second, an empirical link has been established between a country's economic status, as measured by its income, and its energy consumption patterns, industrial activities, and CO_2_ emissions. Developed or high‐income nations typically have different CO_2_ emission profiles than low‐income nations. This disparity is primarily attributable to differences in industrialization, urbanization, and energy consumption levels. Third, employing the World Bank's income classification permits the examination and comparison of CO_2_ emission trends among nations with comparable economic backgrounds, thereby enabling a more nuanced analysis. Fourth, this classification can provide a fundamental framework for the segmentation of our data, as it enables the analysis of CO_2_ emissions at various levels of analysis, ranging from the comparison of emissions among different income groups to a more in‐depth examination of specific national patterns within each group. Finally, this classification is relevant because it captures global economic transitions and shifts that have direct effects on CO_2_ emissions. This is due to the fact that this classification utilizes thresholds to categorize countries, thereby maintaining a consistent standard for measuring development.

## 3 Results

### 3.1 Dependency Between CO_2_ Emissions Indicators

**Figure 1:**
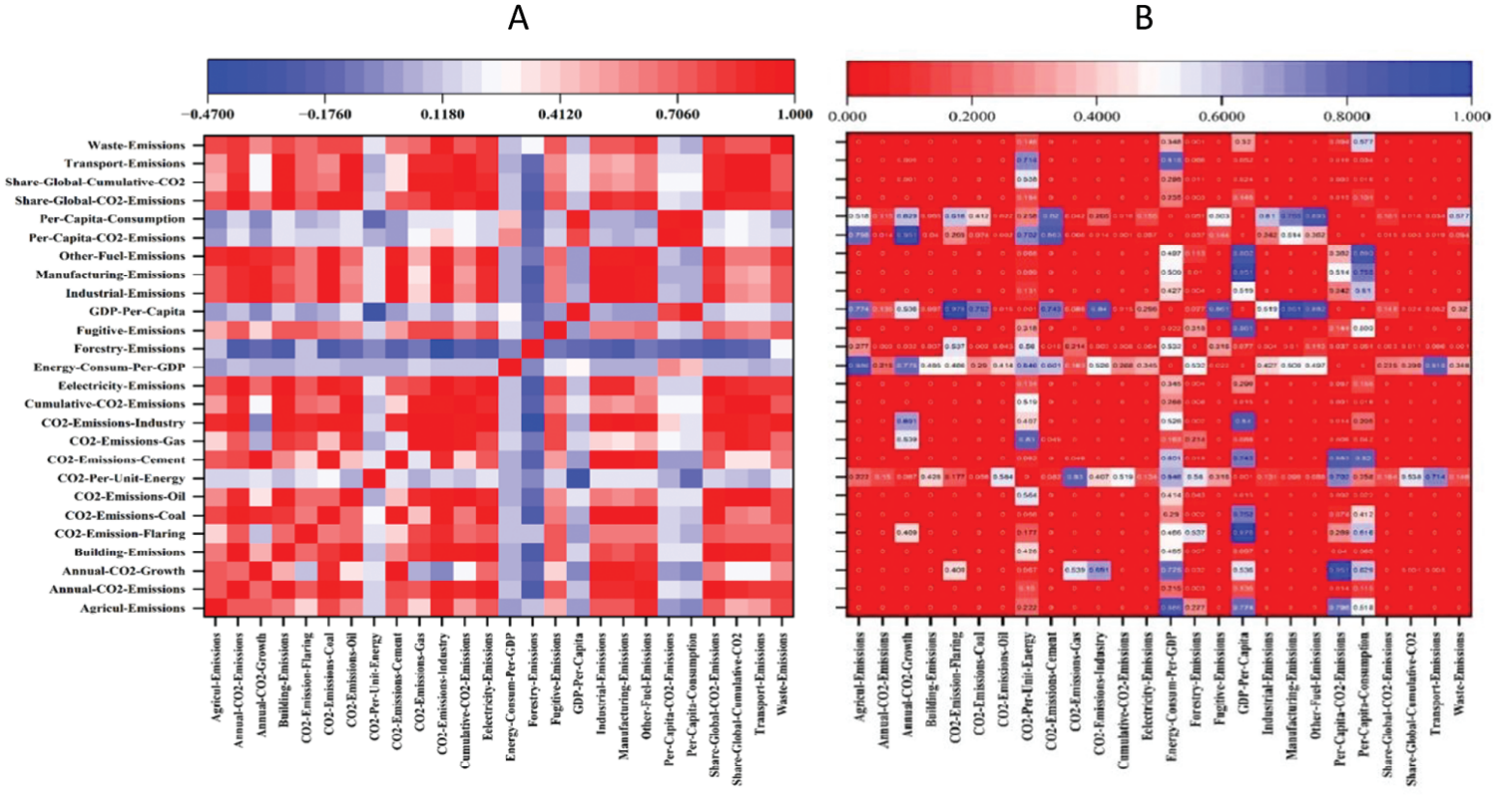
A) Pearson correlation matrix between the 26 indicators, and b) their p ‐ value. The dotted circle in the right panel denote statistically significant correlations at the 95% level (*p* < 0.05).

Prior to running the PCA, we checked for data multidimensionality and colinearity by inspecting the interdependency of the selected 26 indicators. The Kaiser–Meyer–Olkin (KMO) sampling adequacy index showed a value of 0.77, revealing that the chosen indicators are closely interconnected. This implies that a significant portion of the variability among these indicators is due to shared variance. Given the KMO value, the relationships between variables are fairly strong, indicating that PCA will yield clear and reliable components. The Pearson correlation coefficient further supports this interdependence among the indicators, as illustrated in **Figure** **1**. It is noteworthy that the majority of indicators showed positive and statistically significant (*p* < 0.05) correlations, such as between global CO_2_ emissions, cumulative CO_2_ emissions, industrial emissions, waste emissions, transportation emissions, per capita CO_2_ emissions, manufacturing emissions, coal emissions, and building emissions. Emissions from sources like transportation, power generation, industry, gas and oil production, and construction all contributed positively and significantly to the total amount of CO_2_ released into the atmosphere. In addition, industrial and energy production accounted for the bulk of CO_2_ emissions from the burning of fossil fuels. Emissions from manufacturing and industry, gas and coal, annual CO_2_ growth, and other fuels all had significant positive correlations with the global share of emissions. However, in a few cases, such as between forestry emissions and GDP per capita, consumption per capita, CO_2_ emissions per capita, energy consumption per capita, and CO_2_ per unit energy, correlations were negative (and in some cases, even statistically non‐significant). However, in other cases, there were positive and substantial correlations between some of these factors. For example, we noted a positive and statistically significant correlation between GDP‐Per‐Capita and both per capita consumption and per capita CO_2_ emissions.

### 3.2 PCA Results

**Figure 2:**
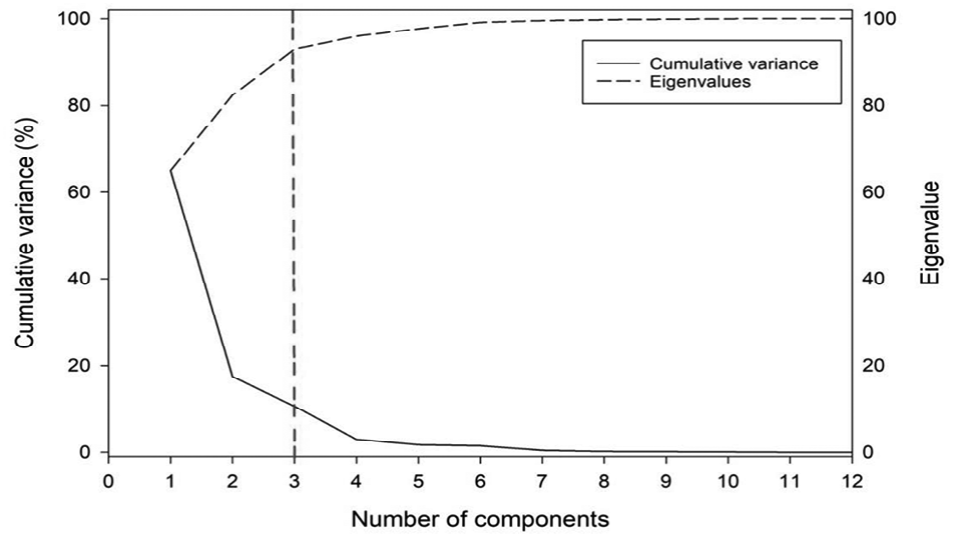
Scree plot justifying retaining the most significant factors that explain the majority of variance in CO2 emission indicators.

In this study, a PCA was applied to the 26 indicators of CO_2_ emissions at the country level. The most significant spatial patterns of CO_2_ emission behavior on the global scale were delineated, where countries assigned to each component (group) exhibit a similar pattern of CO_2_ emission. According to PCA results, three components were retained, which explain together 93% of the total variance in CO_2_ emission data (**Figure** **2**). The first component (PC1) explained alone 40.1% of the explained variance, compared to 37.4% and 15.5% for the second (PC2) and third (PC3) components, respectively. The different CO_2_ emission indicators were assigned to each component according to their loadings (correlations) with the different components. Following this procedure, PC1 denotes the main attributes of CO_2_ emissions, including indicators like share of global cumulative CO_2_, share of global emissions, cumulative CO_2_ emissions, CO_2_ emissions from oil sector, annual CO_2_ emissions, and emissions from the waste sector. On the other hand, some indicators, such as annual CO_2_ growth, and emissions from key sectors like industry, agriculture, and fuel sector, were assigned to PC2, which mainly defined the main sources of the emissions. PC3, which explained only 15.5% of the variance, was best correlated with specific indicators like GDP per capita and CO_2_ emissions per capita, denoting the socioeconomic dimensions of the emissions. To facilitate comparison between the three retained (significant) components, we focused, in further analysis steps, on representative indicators of each component. In this regard, we selected the best correlated two indicators with each component: i.e., cumulative CO_2_ emissions (*r* = 0.96) and share of global cumulative CO_2_ (r = 0.96) for PC1, annual CO_2_ growth (*r* = 0.98) and industrial emissions (*r* = 0.89) for PC2 and GDP per capita (*r* = 0.95) and CO_2_ emissions per capita (*r* = 0.95) for PC3.

### 3.3 Spatial Patterns of CO_2_ Emission: Global, Regional, Versus National Scales

**Figure 3:**
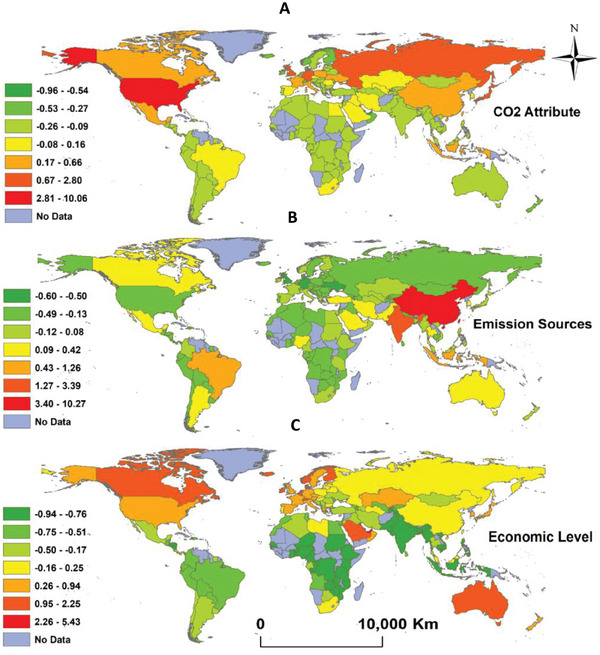
Spatial distribution of the scores of the three different components.

**Figure** **3** depicts the spatial distribution of scores for the three significant components (PC1‐3) that were retained. As illustrated, scores tend to exhibit different spatial variability among the different components. In particular, PC1 (CO_2_ attributes) showed a heterogeneous spatial distribution, with higher values clustered in the developed countries of North America, Europe, Japan, and Russia, and to a lesser extent, China. Rather, PC1 score values were substantially lower in the world's developing regions. PC2 (emissions sources) scores, on the other hand, were most concentrated in East and Southeast Asia, especially in China, India, and Indonesia, in addition to Brazil. Interestingly, for this component that characterizes primarily the annual growth in CO_2_ emissions and output from major sources like manufacturing, farming, and the energy sector, the developing world in Europe and North America scored similarly to the global south. For PC3, which is related more to indicators like GDP per capita and per capita CO_2_ emissions, higher scores were assigned to high‐income countries like Canada, the Gulf countries, Australia, and some Scandinavian countries. Expectedly, developing countries showed lower scores on this component scale, particularly the sub‐Saharan Africa.

**Figure 4:**
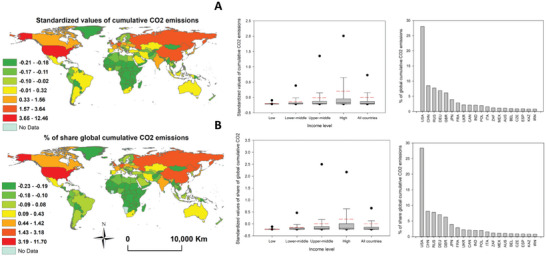
A) Standardized values of cumulative CO_2_ emissions, b) shared global CO_2_ (lower), and their variations amongst low‐, low‐middle‐, upper‐middle‐, and high‐income countries. The high‐ranking countries contributing to the two indicators are also illustrated. The presented two indicators correspond to the best‐correlated variables with the first component (PC1). For the boxplots, the red line represents the mean, and the black line denotes the median. The 10th, 25th, 75th, and 90th percentiles are represented by the horizontal lines, respectively.

**CC**To has more inspections in the regional and country‐level variations in the different CO_2_ emission indicators, we presented the standardized values of the best‐correlated indictors to each component. For PC1, the standardized values of cumulative CO_2_ emissions and the share global cumulative CO_2_ are illustrated in **Figure** **4**. As depicted, for both the cumulative CO_2_ emissions and share global cumulative CO_2_, the most anomalous positive values were typically found in the developing countries in North America, Europe, and eastern Asia. In contrast, it is evident that the global south contributed less to the global cumulative CO_2_ emissions and share global cumulative CO_2_. This picture is reflected when assessing global cumulative CO_2_ emissions and share global cumulative CO_2_ as a function of the economic level of countries. Notably, as a country develops economically, it emits a greater proportion of the world's total CO_2_ emissions and contributes more to the global share. Figure 4 indicates that, over the past three decades (1990‐2016), the US contributed alone to 28.07 and 28.36% of the global cumulative CO_2_ emissions and share global cumulative CO_2_, respectively, followed by China and Russia, which contributed together more than 15% of the global cumulative CO_2_ and share global cumulative CO_2._ More than half of all cumulative CO_2_ emissions come from just five countries: the US, China, Russia, Germany, and the UK.

**Figure 5:**
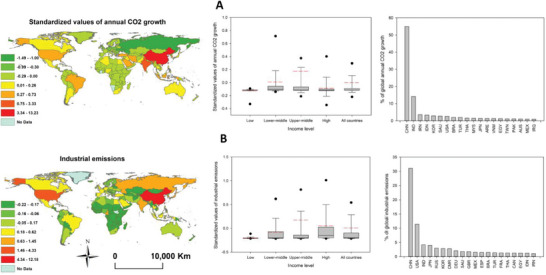
A) Standardized values of annual CO2 growth, b) industrial emissions, and their variations amongst low‐, low‐middle‐, upper‐middle‐, and high‐income countries. The high‐ranking countries contributing to the two indicators are also illustrated. The presented two indicators correspond to the best‐correlated variables with the second component (PC2). For the boxplots, the red line represents the mean, and the black line denotes the median. The 10th, 25th, 75th, and 90th percentiles are represented by the horizontal lines, respectively.

The standardized values of annual CO_2_ growth and industrial emissions, both are representative indicators of PC2, are illustrated in **Figure** **5**. Results indicate that lower‐middle and upper‐middle countries of the world contributed more to annual CO_2_ growth than low‐ and high‐income countries. This is especially evident for China, India, Iran, South Korea, Saudi Arabia, and Brazil, which together accounted for 81.79% of the annual CO_2_ growth in the world between 1990 and 2016, with China contributing alone to more than half (55%) of this increase. A different picture emerges when we focus on industrial emissions, with high‐income countries clearly being the primary contributors to these emissions, followed by upper‐middle‐income countries. The industrial emissions of countries with low and low‐middle‐income levels were, as expected, lower. Only five countries (China, the United States, India, Japan, and Russia) accounted for over 53% of global industrial emissions from 1990 to 2016. Amongst them, China was responsible for emitting more than 30% of the global industrial emissions, followed by the USA (10%). Interestingly, among the top 20 countries responsible for industrial emissions worldwide over the past few decades were several developing nations, including Saudi Arabia (1.84%), Egypt (1.22%), Indonesia (1.21%), and Iran (1.05%).

**Figure 6:**
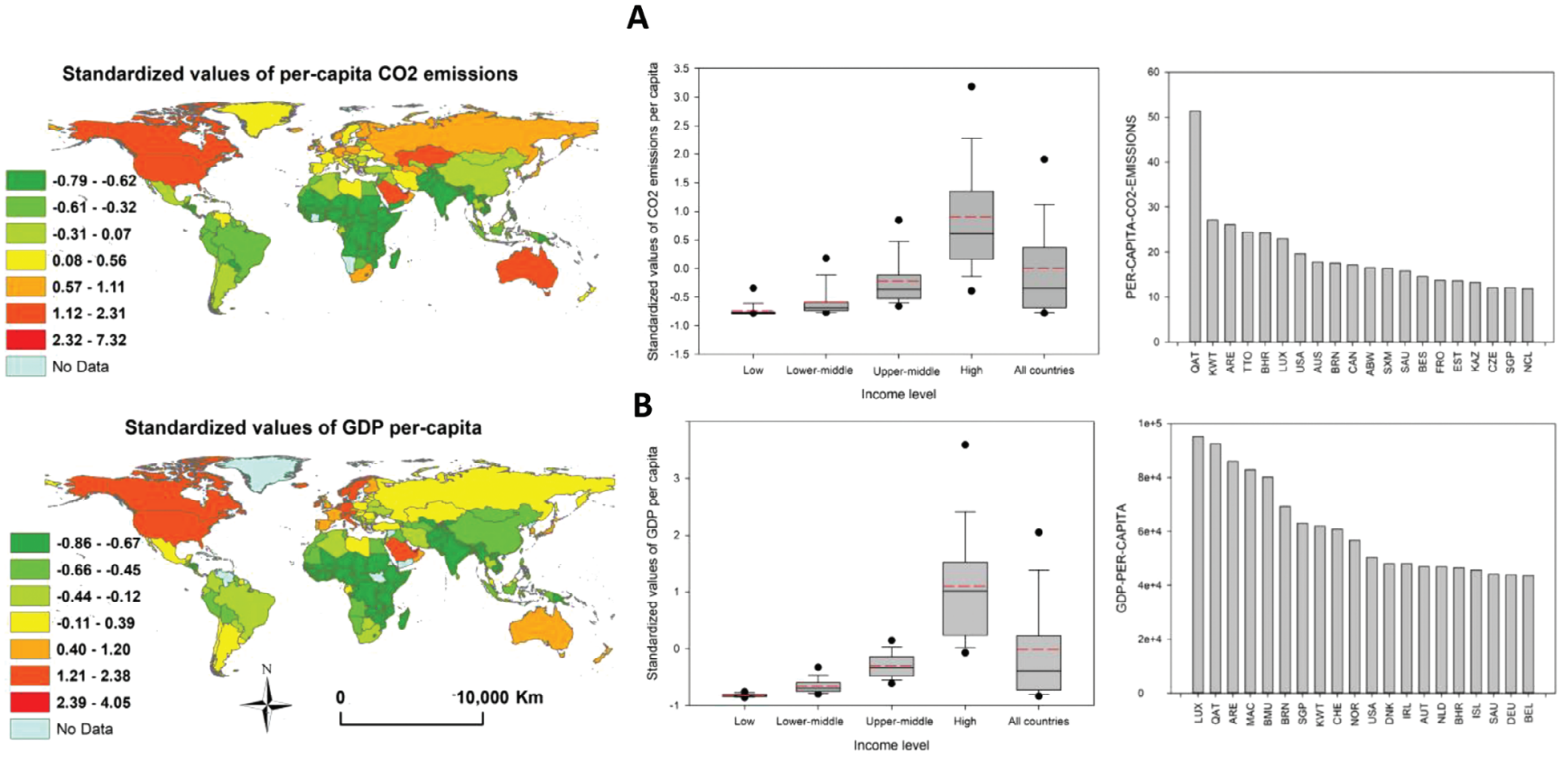
A) Standardized values of per‐capita CO_2_ emissions, b) and GDP per‐capita (lower), and their variations amongst low‐, low‐middle‐, upper‐middle‐, and high‐income countries. The high‐ranking countries contributing to the two indicators are also illustrated. The presented two indicators correspond to the best‐correlated variables with the third component (PC3). For the boxplots, the red line represents the mean, and the black line denotes the median. The 10th, 25th, 75th, and 90th percentiles are represented by the horizontal lines, respectively.

**Figure** **6** illustrates the standardized values of two indicators, per‐capita CO_2_ emissions, and GDP per‐capita, representing PC3. The standardized values are presented for the globe and as a function of the income level. Notably, the standardized values of both indicators show clear differences as a function of income level, with positive anomaly being clearly found for high‐income countries in comparison to those with lower incomes. Total CO_2_ emissions appear to be positively correlated with per capita income. At the country level, it was found that the Gulf states (including Qatar, Kuwait, Bahrain, and Saudi Arabia), with their oil‐based economies and high GDP per capita, had the highest levels of per‐capita CO_2_ emissions.

### 3.4 Temporal Patterns of CO_2_ Emissions Indicators

**Figure 7:**
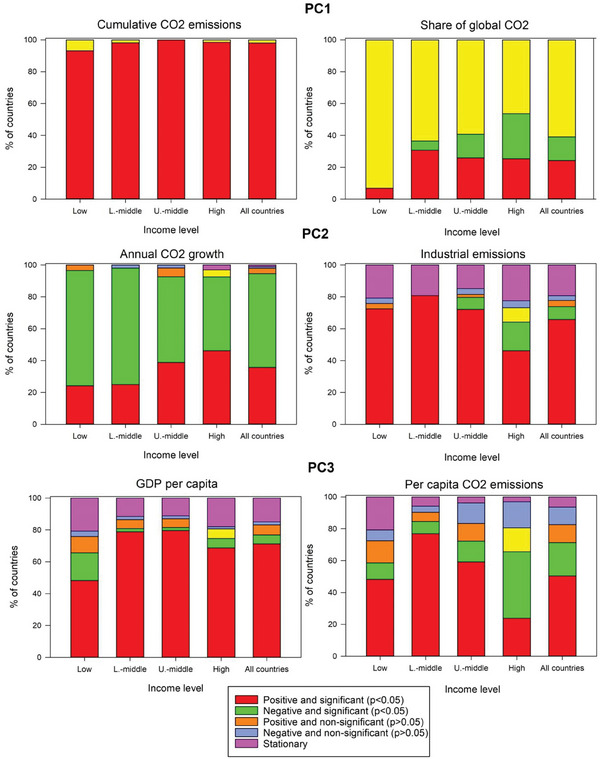
Significance of changes in the selected indicators for the period 1990–2016. Statistical significance was assessed using the modified Mann–Kendall statistic at the 95% level (p < 0.05).

The modified Mann‐Kendall test at the 95% confidence interval was used to analyze trends in the selected CO_2_ emissions from 1990 to 2016. The statistical significance of the trends was evaluated as a function of a spectrum of national income levels. **Figure** **7** shows that irrespective of the income level, a dominant statistically significant increasing trend (*p* < 0.05) in cumulative CO_2_ emissions was observed, with over 90% of countries exhibiting this positive trend. More than half of the world's countries showed stationary behavior and less variability in their share of global CO_2_ between 1990 and 2016. In the meantime, other countries' share of global CO_2_ was statistically significant positive and negative trends. In particular, excluding low‐income nations, nearly 25% of all countries worldwide showed a statistically significant upward trend in their share of global CO_2_. Conversely, significant negative trends were noted for 5.80, 14.81, and 28.36% of the lower‐middle, upper‐middle, and high‐income countries, respectively.

For indicators corresponding to PC2, the majority of countries showed a negative and statistically significant trend in annual CO_2_ growth. There was a statistically significant difference between the world countries, given that a higher number of low‐middle (73.08%) and low‐income countries (72.4%) exhibited a negative trend in the annual CO_2_ growth, compared to upper‐middle (53.7%) and high‐income (46.27%) countries. In contrast, a dominant positive trend in industrial emissions was noted, regardless of the income level, though a larger share of emerging economies (i.e., low, lower‐middle, and upper‐middle countries) than high‐income countries (46.27%) was found. Notably, no income group witnessed more than a 5% reduction in industrial emissions between 1990 and 2016.

The GDP per capita indicator showed significant and positive trends over the past three decades (Figure 7). However, this increase was much higher for the lower‐middle (78.85%) and upper‐middle (79.63%) countries than for the low (48.28%) and even high‐income (68.66%) countries. We found that low, lower‐middle, and upper‐middle‐income countries had the strongest positive trends in CO_2_ emissions per capita. While nearly 41% of high‐income countries showed a significant decrease in their CO_2_ emissions per capita, only 23.89% showed a significant increase.

**Figure 8:**
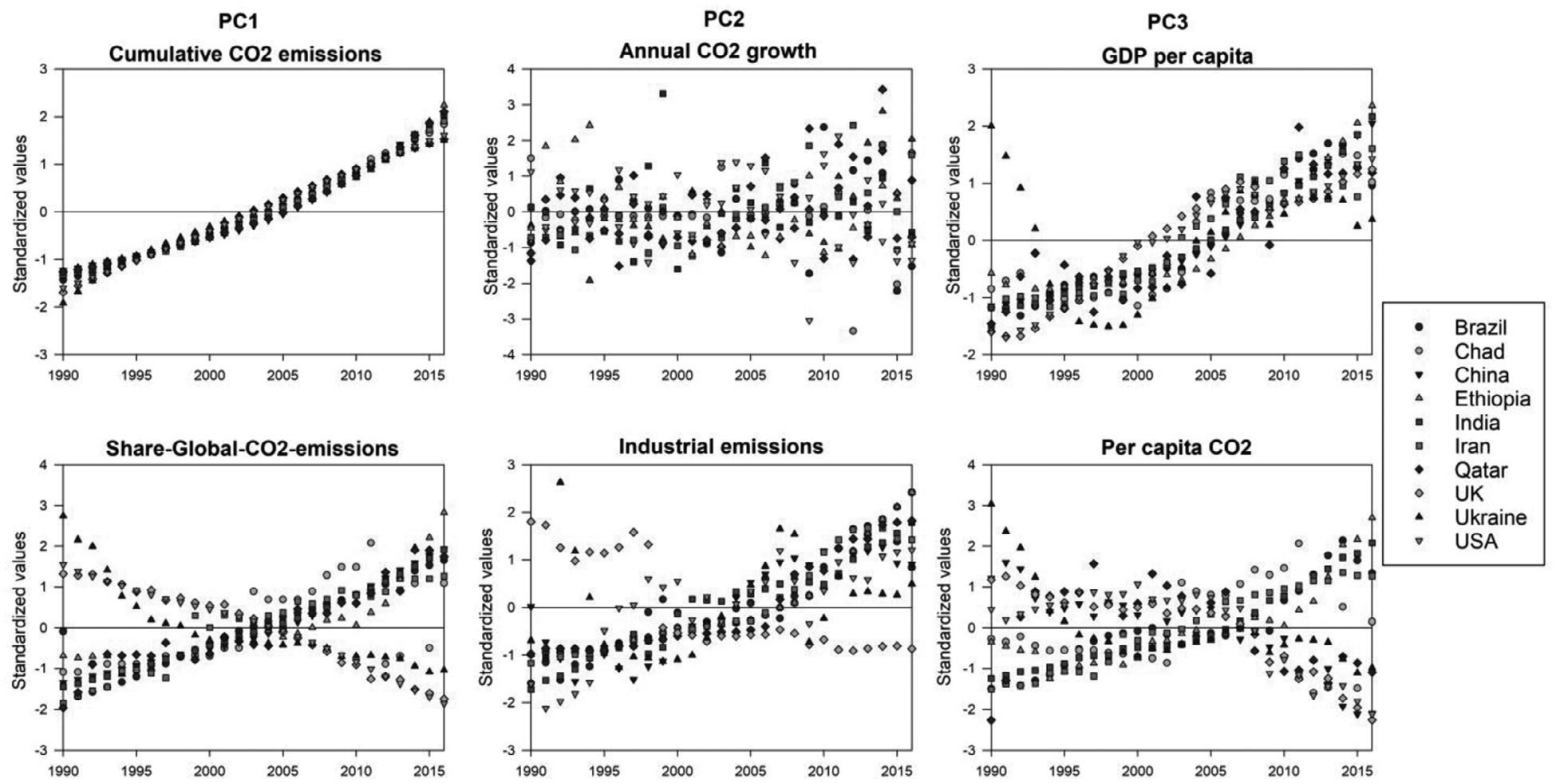
Temporal variability of the different CO_2_ emissions indicators for the selected ten countries between 1990 and 2016. The standardized anomalies of each timeseries are plotted to facilitate direct comparison between different indicators, as well as different countries.

In order to explore how various countries are experiencing such divergent trends in their CO_2_ emissions, ten countries, representing a range of income levels and geographic locations, were chosen for a more in‐depth examination of their CO_2_ emission indicators' trends between 1990 and 2016. These countries are Ethiopia and Chad from low‐income countries; Ukraine, Iran, and India from lower‐middle‐income countries; China and Brazil from upper‐middle‐income countries; and the United States, Qatar, and the United Kingdom from high‐income countries. Our goal is to examine geographically how these indicators vary across nations, regions, and continents. The results of temporal variability of CO_2_ emissions in these countries are illustrated in **Figure** **8**.

Figure 8 displays distinct rising trends in cumulative CO_2_ emissions across the selected ten countries, with almost a steady annual increase. This was evident for all countries, albeit with more exponential growth in CO_2_ emissions for some low‐, lower‐, and upper‐middle‐income countries, such as Ethiopia, Chad, and Iran. For the share of global CO_2_ emissions, we identified two contradictory patterns, with a remarkable decline in high‐income countries like the US and the UK, while a positive trend was seen for low‐middle (e.g., India) and upper‐middle (e.g., China and Brazil) countries.

For the annual CO_2_ growth, most of the countries shared a slowing trend in annual CO_2_ growth, especially from 2000 to 2009. Wealthy nations such as the US, Qatar, and the United Kingdom are prime examples of this pattern. For other countries like India and China, the annual CO_2_ emissions increased rapidly over time. A deep inspection of this figure reveals that high‐income countries produce more CO_2_ each year. This is clearly confirmed when looking at changes in the industrial emissions, where two patterns of change can be observed. Albeit with the overall rising levels of industrial emissions in all selected countries, apart from the UK, some countries like Ethiopia, Chad, Iran, India, and Qatar experienced consistent growth, while other countries witnessed more erratic growth (e.g., Ukraine).

As illustrated in Figure 8, the GDP per capita has increased over time for almost all countries, although many countries exhibited a steady behavior in their GDP per capita during the Great Recession of 2008–2010. Furthermore, it is noted that some countries with high per capita income, such as the US, the United Kingdom, and Qatar experienced the most dramatic changes in their GDP‐Per‐Capita over the past decade. As depicted in Figure 8, high‐income countries like the US, UK, and Qatar exhibited a significant decrease in their per capita CO_2_ emissions during the last decade. Conversely, other countries, like Ethiopia, India, and China, witnessed a steady and continuous increase in their per capita CO_2_ emissions, which followed the same pattern of growth in their GDP per capita.

### 3.5 Main Contributors to Global CO_2_ Emissions

**Figure 9:**
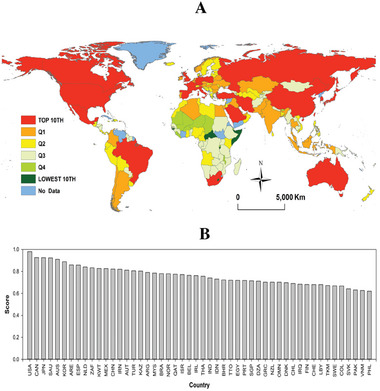
A) Spatial distribution of the overall composite score summarizing the main contributors to global CO2 emissions and divided into different categories, and b) the rank of the top country‐contributors. In panel a, Q1 is occupied by the top 25% of countries on the list; Q2 represents countries in the 25% to 50% range; Q3 shows countries in the 50 to 75% range; and Q4 is occupied by countries in the 75% to 100% range. The top 10th contributors and the lowest 10th emitters are also mapped.

In an attempt to provide a complete picture of the main countries contributing to global CO_2_ emissions, we calculated an aggregated score index that takes into account the relative contributions of each country to all individual indicators (Section 2.2). The main country contributors to the global emissions of CO_2_ and their spatial patterns are depicted in **Figure** **9**. The world's rising emissions can be traced back to a list of few countries; most of them are either highly‐developed nations (e.g., the US, Japan, China) or major oil exporters (e.g., Saudi Arabia, Kuwait, Bahrain, Qatar, Libya, Iraq, and Oman), or emerging economies (e.g., Brazil, Mexico, Singapore, Turkey, and Pakistan). Notably, between 1990 and 2016, some of the most developed EU members (e.g., France and Germany) were not listed among the largest contributors. In contrast, some countries like Spain, Netherlands, Norway, and Portugal were the main contributors.

**Figure 10:**
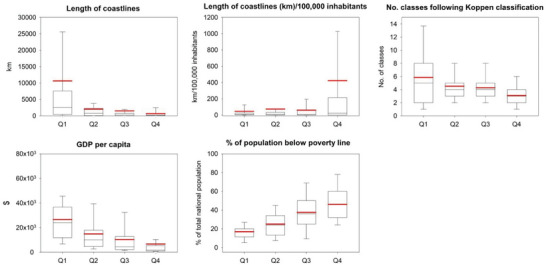
Scatterplots showing differences in a set of environmental and socioeconomic variables as a function of the four quartiles of the world countries, which were defined based on their contribution to global CO_2_ emissions. The total length of the coastline, the length of coastline per population (km/100 000 inhabitants), and the number of climate classes following the Koppen climate classification were employed as indicators of the physical environment of the countries, while GDP per capita and percentage of national population below the poverty line were presented as indicators of their socioeconomic environments. For each box plot, the red line represents the mean, and the black line denotes the median. The 10th, 25th, 75th, and 90th percentiles are represented by the horizontal lines, respectively. Data regarding coastline length, population, GDP per capita, and the number of individuals below the poverty line were sourced from the Encyclopedia of the Nations (retrieved from https://www.nationsencyclopedia.com/ on September 25, 2023).

Based on AI‐derived data on their global CO_2_ emissions contributions, countries have been categorized into four primary groups: Q1 encompasses the top 25% of countries on the list, Q2 including countries in the 25–50% bracket, Q3 covering countries ranging from 50%–75%, and Q4 representing countries in the 75–100% bracket (Figure 9). The legitimacy of these groupings is reinforced when considering the disparities in environmental and socioeconomic conditions among countries within these quartiles. **Figure** **10** illustrates the variations observed in important metrics. As illustrated in Figure 10, the proximity to coastal regions seems to play a significant role in determining a nation's CO_2_ emissions. Countries that are major contributors to emissions (Q1 and Q2) often possess extensive coastlines. For example, countries classified as Q1 exhibit an average coastline length exceeding 10,000 km, whereas Q4 countries, which make comparatively smaller contributions, display an average coastline length of approximately 670 km. This observation is further substantiated when analyzing population densities along these coastal regions. In countries characterized by high levels of emissions, it can be observed that approximately 46.5 km of coastline is occupied by every 100000 inhabitants. On the contrary, within nations categorized as Q4 with lower emission levels, a comparable population is distributed along approximately 420 kilometers of coastline. Another noteworthy environmental factor to consider is the prevailing climatic conditions, as categorized by the Koppen classification system. As depicted in Figure 10, nations characterized by significant CO_2_ emissions typically exhibit a more extensive range of climate classifications, thereby suggesting a heightened degree of climatic diversity in comparison to other countries with low emissions.

The analysis of socioeconomic indicators, such as GDP per capita and the proportion of the population living below the poverty line, reveals discernible patterns, as depicted in Figure 10. For Q1, countries exhibit an average GDP per capita of approximately $26000, indicating an elevated economic status. On the other hand, the nations in the second, third, and fourth quarters exhibited GDP per capita figures of approximately $15 000, $10 000, and $6500, respectively. Moreover, upon examining the segment of the population residing below the poverty line threshold, it becomes evident that countries of Q4 witnessed a remarkable poverty rate of 45.9%, closely trailed by Q3 with a poverty rate of 37.5%. In contrast, countries of Q1 and Q2 exhibited comparatively lower proportions, standing at 17% and 25% respectively. These findings underscore that countries with higher CO_2_ emissions, namely Q1 and Q2, generally have a more robust economic position and a smaller proportion of their population in poverty compared to the lower‐emitting countries in Q3 and Q4.

## 4 Discussion

The rising emission of GHGs has become a major international concern, due to its adverse impacts on both natural systems and human environments.^[^ ^35^, ^36^, ^37^ ^]^ Amongst GHGs, CO_2_ emissions dominated all other types over the past decades, inducing several negative impacts such as melting of snow and ice, an increase in the global average sea level, and an increase in extreme climate events such as drought, heavy precipitation, and tropical cyclones.^[^ ^38^, ^39^, ^40^, ^41^ ^]^ The present study aimed to investigate the spatial and temporal distribution of CO_2_ emissions, based on a set of 26 indicators, at various scales including global, regional, and national over the period from 1990 to 2016. PCA was used to simplify our dataset by reducing its dimensions, making interpretation and analysis easier. This method also addresses the high colinearity among indicators, identifying the dominant global spatial patterns of CO_2_ emissions. Furthermore, by utilizing the World Bank's classification of countries according to their income levels, we provided a more comprehensive understanding of the characteristics and factors influencing CO_2_ emissions in different economic settings.

The primary objective of our analysis was to condense the 26 indicators of global CO_2_ emissions into a limited number of principal components through PCA. The study generated a KMO value of 0.77, indicating that the selected indicators are ideal for PCA and making them promising to produce clear and reliable components. The PCA suggested a set of three primary components adequately accounted for a remarkable 93% of the variability observed in the data pertaining to CO_2_ emissions. The results provided a thorough depiction of various themes, including the characteristics of emissions (PC1), their main origins (PC2), and their socio‐economic implications (PC3). This categorization implies a significant level of data compression and reduction in dimensionality while preserving the fundamental content of the initial information. As indicated, each individual component appeared to symbolize a distinct “theme” or pattern within the dataset, providing evidence on the efficacy of the PCA method in delineating the different attributes of CO_2_ emissions. Specifically, the initial principal component (PC1) encapsulated significant characteristics of CO_2_ emissions, such as the cumulative amount of CO_2_ emitted and the yearly emission levels. This component serves as a representation of the inherent characteristics of emissions, offering valuable information regarding the combined and yearly levels of CO_2_ emissions, thereby illuminating the scope and pace of emissions on a worldwide basis. The second principal component (PC2) primarily delineated the primary sources of emissions, including industry and agriculture. This component classifies the primary sources of emissions, with a particular emphasis on industry and agriculture, as these sectors are frequently identified as the primary contributors.^[^ ^8^, ^42^, ^43^, ^44^ ^]^ This component facilitates the identification of sectors that may require interventions or more rigorous regulatory measures. In the interim, the third component (PC3) represented the socio‐economic aspects of emissions, emphasizing indicators such as per capita GDP and per capita CO_2_ emissions. This component underscores the socio‐economic aspects of emissions, establishing a connection between economic progress and environmental consequences. It places particular emphasis on variables such as per capita GDP and CO_2_ emissions, suggesting the utmost importance of comprehending the interactions between economic growth and environmental sustainability. This association agrees well with one of the most frequently referenced theories in the field of environmental economics: the Environmental Kuznets Curve (EKC) hypothesis.^[^ ^45^ ^]^ This hypothesis posits a relationship between economic development and environmental degradation. The proposition posits that as economic development advances, there is a corresponding increase in environmental degradation, albeit limited to a specific threshold. This curve has been employed in academic research to examine the correlation between GDP and a range of pollutants, encompassing CO_2_.^[^ ^46^, ^47^ ^]^ Importantly, the socio‐economic implications highlighted by PC3 underscore the balance between economic prosperity and environmental responsibility. Overall, the arrangement of scores associated with the three retained key components provides a comprehensive perspective on worldwide CO_2_ emissions, highlighting the intricate relationship between economic advancement, industrial growth, and their environmental consequences.

The PC1 encapsulates fundamental characteristics of CO_2_ emissions. Primarily, developed nations located in regions such as North America, Europe, Japan, Russia, and China exhibit elevated scores, providing evidence of their prominent position in global CO_2_ emissions. The emergence of this phenomenon can be attributed to the advent of industrialization, during which countries like the US, the UK, and Germany experienced rapid economic expansion, albeit at the cost of the environment. This historical progression highlights the ongoing discourse surrounding the responsibilities of nations that played a leading role in the process of industrialization, thereby contributing significantly to the accumulation of greenhouse gases in the Earth's atmosphere on a global scale. The primary emphasis of PC1 is on recognizing the historical accountability of these nations, as their prosperity came at the cost of adversely influencing global climate patterns.^[^ ^48^, ^49^ ^]^

PC2 explores the analysis of the origins and pathways of emissions. Both established and emerging economies have made significant contributions, highlighting the global scope of the challenge. Countries such as China, India, and Brazil have experienced industrial advancements that have led to an increase in their emissions. This trend mirrors the growth trajectories observed in developed nations, but these emerging economies have placed an additional emphasis on sustainability. Within this particular framework, the notable emissions originating from countries such as China, India, Iran, South Korea, and Brazil, particularly during the period spanning from 1990 to 2016, underscore the imperative for international cooperation, pioneering environmentally friendly technologies, and a fundamental transformation in industrial methodologies to address and alleviate the rapid increase in emissions.^[^ ^50^, ^51^, ^52^ ^]^

PC3 examines the relationship between a country's level of economic prosperity and its emissions of carbon dioxide. A discernible correlation arises, particularly in developed and oil‐rich countries, where strong economic growth frequently coincides with increased levels of emissions. For example, nations such as Qatar and Saudi Arabia, which possess significant oil reserves, have observed a clear correlation between their economic prosperity derived from fossil fuel resources and the subsequent rise in CO_2_ emissions. The conclusions drawn from our analysis align with the research conducted by Aslam et al.^[^ ^53^ ^]^ and Magazzino et al.,^[^ ^54^ ^]^ which highlight the significant role played by GDP growth and the increasing share of industry's GDP in driving the upward trajectory of CO_2_ emissions. The metric of GDP per capita is frequently used to establish a connection between economic growth and environmental impact. This association has been observed in various recent studies, such as Chen et al.^[^ ^55^ ^]^ who examined the various factors influencing China's CO_2_ emissions between 1995 and 2012. The results of our study indicate that there has been a substantial decrease in GHG emissions within the European Union (EU) between the years 1990 and 2016. The observed trend can be primarily attributed to a transition towards a service‐oriented economy, as well as improvements in energy efficiency.^[^ ^56^, ^57^, ^58^, ^59^ ^]^ According to González‐Sánchez and Martín‐Ortega,^[^ ^60^ ^]^ the EU experienced a decrease of 23.45% in GHG emissions during the period from 1990 to 2017. Additionally, there was a marginal rise in the energy sector's contribution to these emissions, increasing from 77% to 79%. The decrease in energy consumption was made possible through the implementation of improved energy efficiency measures, the adoption of greater proportions of renewable energy sources, and the implementation of policies such as the European Union's Green Deal, which seeks to promote sustainable development and the advancement of environmentally friendly technologies. Multiple scholarly investigations have underscored the noteworthy contribution of renewable energy sources in attaining this accomplishment.^[^ ^61^, ^62^, ^63^, ^64^ ^]^ Nonetheless, countries heavily reliant on coal, such as Poland and the Czech Republic, present significant obstacles to the European Union's aspirations of achieving carbon neutrality. Therefore, it is imperative to prioritize the development and implementation of innovative technologies that facilitate the transition from coal to more environmentally friendly alternatives.^[^ ^65^, ^66^ ^]^ In contrast, the Gulf Cooperation Council (GCC) region exhibits a distinct situation. Despite undertaking eco‐modernization initiatives such as Abu Dhabi's Masdar City and Dubai's Green Building Code,^[^ ^67^, ^68^ ^]^ the pursuit of decarbonization in these regions is hindered by the presence of lower fuel prices. The pricing policy implemented has had a negative impact on the promotion of efficient energy usage, resulting in limited integration of renewable energy sources within their economies.^[^ ^61^, ^69^, ^70^ ^]^ Nevertheless, it is widely argued by scholars that the GCC possesses the capacity to efficiently exploit renewable energy sources such as wind, solar, and hydropower.^[^ ^2^, ^71^, ^72^, ^73^, ^74^ ^]^ Overall, the clear correlation between economic growth and emissions underscores the necessity for global strategies that prioritize the dual objectives of economic and environmental sustainability. According to van den Bergh,^[^ ^75^ ^]^ there is a need for a paradigm shift that promotes both economic prosperity and the preservation of the planet's ecological equilibrium.

In order to effectively combat global warming, it is crucial to grasp how various countries are experiencing divergent trends in their CO_2_ emissions. Althor et al.^[^ ^17^ ^]^ observed that many of the highest‐emitting countries are less vulnerable to the potential consequences of climate change. On the other hand, several nations with relatively low GHG emissions show significant susceptibility to the detrimental effects of climate change. In our study, differences between countries, with different economic levels, in their CO_2_ emission evolution were assessed. Specifically, we compared changes in CO_2_ emission for selected ten countries, representing various levels of economic growth and located at different continents. This assessment can give insights into the main drivers of CO_2_ emissions growth. Findings indicate that GDP and industrial emissions are primary indicators of GHG emissions worldwide, with the most pronounced increase amongst all indicators. The percentage change in industrial emissions from 1990 to 2016 varied widely from country to another, ranging from a decrease of −63.21% in the UK to an increase of 185.79% in Ethiopia (**Table** **2**). Even among the most developed economies, this rate varied widely: 139.08% in China and 18.07% in the US. A similar pattern was evident for GDP per capita. Different economic, demographic, climatic, and behavioral characteristics of each country can account for the variations in CO_2_ emissions change for the period 1990–2016. Knowledge of the main factors determining CO_2_ emission growth and differences in the impact of these factors at country level is of utmost importance and necessitates further investigation to improve the design and implementation of nationally appropriate mitigation actions. This is simply because the effectiveness of actions to reduce CO_2_ emissions depends largely on the characteristics and evolution of the national economy and national emission sources.

**Table 2:** Percentage of change in CO_2_ emission indicators during 1990‐2016 for the selected 10 countries.

	PC1		PC2		PC3	
	Cumulative CO_2_ emissions	Share global CO_2_ emissions	Annual CO_2_ growth	Industrial emissions	GDP per capita	Per Capita CO_2_ emissions
Brazil	4.56%	17.80%	4.95%	36.36%	24.31%	18.48%
Chad	4.32%	29.38%	25.21%	0.01%	41.33%	21.76%
China	4.37%	18.41%	18.67%	139.08%	125.50%	18.92%
Ethiopia	4.37%	33.03%	41.77%	185.79%	81.04%	30.96%
India	4.62%	11.48%	12.87%	100.03%	77.84%	12.65%
Qatar	4.25%	35.96%	34.77%	177.51%	3.64%	9.37%
UK	7.07%	‐423.93%	3.96%	‐63.21%	23.27%	‐19.52%
Ukraine	5.12%	13.91%	‐0.03%	15.11%	14.31%	12.02%
USA	5.87%	10.90%	29.61%	18.07%	24.06%	10.41%

Our results demonstrate that the global CO_2_ emissions are distributed very inequitably (Figure 9).

The distribution of global CO_2_ emissions exhibits a significant imbalance, with a predominant contribution originating from developed nations such as the US, Japan, South Korea, and Spain, as well as GCC countries and emerging economies like Mexico and Brazil. China has been identified as a significant contributor to CO_2_ emissions in various studies.^[^ ^76^, ^77^, ^78^, ^79^ ^]^ China's notable emissions can be attributed to several factors, namely the swift process of industrialization, urbanization, economic expansion, heavy dependence on extensive coal reserves, and its status as the global center for manufacturing. Nevertheless, these studies frequently rely on traditional measurements of CO_2_, such as overall emissions, emissions per capita, or emissions intensity in relation to GDP. Although these metrics are valuable, they may fail to consider the intricate elements of a nation's emissions profile. The present study employs a broader range of indicators, thereby offering a more nuanced perspective on the impact of China's emissions. For example, China does not exhibit leadership in certain significant indicators, such as per capita CO_2_ emissions or forest emissions. Moreover, when evaluating China's emissions from a consumption‐based standpoint, which takes into account both imported and exported goods and services, a distinct perspective emerges. This perspective recognizes that a significant portion of China's emissions are associated with products that are consumed in other regions. In this regard, while our findings provide additional support to previous research, it is imperative to gain a comprehensive understanding of the intricate emission patterns in China. China has demonstrated commendable advancements in the adoption of renewable energy sources, technological advancements, and the establishment of policy frameworks aimed at mitigating emissions.^[^ ^80^, ^81^ ^]^ The aforementioned endeavors may not yield immediate outcomes in conventional emission measurements, yet they hold significant importance in comprehending the prospective trajectory of China's carbon dioxide emissions.

The categorization of nations based on their individual levels of contribution to worldwide CO_2_ emissions has become increasingly significant in response to the persistent challenges presented by climate change. The utilization of AI‐derived quartile classification (Section 3.5) offers a feasible method for validating the methodology employed in this study. In this research, nations were assessed and categorized into four primary groups (Q1:Q4) according to their levels of carbon emissions. Significant disparities were identified among the nations encompassed within the four categories, with regards to their physical and socioeconomic environments (Figure 10). These disparities serve as key factors in elucidating the varying degrees of their respective contributions to global CO_2_ emissions. The primary drivers of global CO_2_ emissions were primarily nations characterized by extensive coastlines and significant population concentrations within these coastal areas. Coastal regions have been intricately linked with diverse forms of economic activity. Additionally, ports play a pivotal role in facilitating global trade, serving as vital centers for the exchange of commodities and services. The spatial proximity of a given area to a body of water frequently results in the agglomeration of both human settlement and industrial enterprises. The notable disparity in population density along coastal regions between nations characterized by high and low emissions underscores a noteworthy observation. Urban agglomerations are frequently associated with elevated population densities in coastal areas. The results of our study align with prior research that has underscored the correlation between extended coastlines and the existence of robust port infrastructure, thereby enabling heightened industrial engagement. As exemplified by the findings of Seto et al.^[^ ^82^ ^]^ urban areas located near coastal regions display heightened rates of growth and exhibit increased levels of economic productivity. Furthermore, McGranahan et al.^[^ ^83^ ^]^ proposed that coastal areas often encounter the simultaneous task of adapting to climate change caused by rising sea levels and mitigating its impacts due to significant emissions. Hence, it can be deduced that countries with extensive coastlines may exhibit a higher inclination to make substantial contributions to CO_2_ emissions. In the same context, we utilized the Koppen classification system as a prominent methodology for categorizing the prevailing climatic conditions in various countries across the globe. The findings of our study have demonstrated a significant correlation between diverse climatic conditions and their impact on global CO_2_ emissions. According to Peel et al.^[^ ^84^ ^]^ nations that possess a greater degree of climatic diversity tend to showcase a wider array of industries and agricultural practices. These factors collectively contribute to the overall carbon footprint of a country. The incorporation of poverty rates offers supplementary contextual information to this narrative. The observation of elevated poverty rates in nations with lower emissions may indicate a scarcity of industrial progress, a correlation that aligns with the findings of Raupach et al.^[^ ^85^ ^]^ who noted a significant association between industrial activities and a country's carbon emissions. In conclusion, it is evident that the geographical, climatic, and socioeconomic factors of a country play a substantial role in shaping its carbon footprint. The challenge lies in adeptly navigating these intricate dynamics while advancing toward a more sustainable future.

To conclude, there has been relatively limited literature employing a layered approach to examine the spatial and temporal attributes of CO_2_ emissions, specifically in terms of global versus regional and national scales. In this study, a comprehensive and extensive inventory was developed, encompassing a span of multiple decades and utilizing 26 distinct indicators of CO_2_ on a global scale. The nuanced shifts in these CO_2_ indicators were examined using PCA and trend analysis, allowing for the tracing of their evolution and variability across various geographical scales. Our methodology enables the recognition of broad trends as well as distinct deviations on regional and national scales, which is essential for the formulation of efficient measures to mitigate GHGs emissions. The interpretability and insights provided by the retained principal components are substantial, despite the fact that they represent a condensed version of the initial set of 26 indicators. Overall, a main innovative aspect of this study is its capacity to integrate extensive datasets into coherent themes through PCA, which enhances understanding, formulation of strategic approaches, and implementation of more focused interventions at the global, regional, and national scale. Assessing the spatial and temporal evolution of CO_2_ between continents and regions is necessary for understanding and formulating global CO_2_ emissions reduction policies. In addition, identifying the spatiotemporal characteristics of CO_2_ emissions is a vital task to better achieve CO_2_ reduction targets formulating adequate policies, and define actions to achieve this goal.

Here, it should be emphasized that some uncertainties might be introduced in the obtained results. These uncertainties may be originated from lack of national data for some countries and for specific indicators. Furthermore, in some countries, estimated CO_2_ emissions from a few single sources (e.g., forestry) might not be close to real emissions. However, the percentage of country total emissions that come from these sources is generally negligible. Furthermore, the impact of these uncertainties is minimized recalling that we developed an aggregated indicator, on a scale of 0 to 1, which accounts for a wide variety of CO_2_ indicators. As this indicator relies on numerous global metrics, it can provide aggregated assessments at the national level, which is relevant for international policy negotiations.

## 5 Conclusions and Policy Implications

GHG emissions have been identified as a primary driver of climate change worldwide. CO_2_ emissions have been the primary focus of the available studies on GHG emissions in recent decades, with a focus on determining the main sources and sinks of these emissions. However, to get a full picture of CO_2_ emissions, it is necessary to understand the complexity and multilinearity between the different indicators of these emissions from both spatial and temporal perspectives, which have not been taken into account in previous studies. Our study is dedicated to constructing a long‐timescale CO_2_ emission inventory using 26 indicators on the global scale. The spatial and temporal variability of these indicators at the global, regions, and country‐level scale was investigated using the PCA and trend analysis. PCA suggested three main components, explaining a remarkable portion (93%) of CO_2_ emission variability, spanning the different attributes of these emissions like emissions characteristics (PC1), origins (PC2), and socio‐economic effects (PC3). We focused our analysis on the best correlated two indicators for each component, including indicators like share global cumulative CO_2_ emissions, global cumulative CO_2_ emissions, annual CO_2_ growth, industrial emissions, per‐capita CO_2_ emissions, and GDP per capita. Results demonstrate that the USA is the largest contributor to the global cumulative CO_2_ emissions and share global cumulative CO_2_ emissions, while China exhibited the highest annual CO_2_ growth and industrial emissions. High‐income countries were among the highest contributors to the per‐capita CO_2_ emissions and GDP per capita. Notably, Europe exhibited a significant reduction in their GHG emissions between 1990 and 2016, which are mainly attributed to their evolution of GDP and final energy intensity. In contrast, the decoupling between GDP and CO_2_ emissions were less evident for the GCC countries, which can be linked to the inexpensive energy prices, as well as the low share of renewable energy sectors in these countries.

To improve CO_2_ emissions efficiency and to promote the balance between countries in different income categories, some specific policy implications can be generated from the findings of this study.

‐ The high‐income and upper‐middle‐income as economic leading countries should strengthen the current policies and intervention to formulate and implement more stringent CO_2_ emissions reduction measures, promote the optimization of industrial carbon emissions, and rely more on renewable energy rather than on fossil fuel.
‐ In addition to implementing international agreements, countries should implement differentiated strategies depending on the amount of the CO_2_ emitted by the country when formulating CO_2_ emissions reduction policies.
‐ As many low‐income and lower‐middle‐income countries are in the process of industrial transformation and have a high degree of urbanization and economic development and high demand for environmental improvement and industrial upgrading, these countries are significantly contributing to emitting CO_2_ emissions. Therefore, these countries should formulate and implement stringent CO_2_ emissions reduction and environmental improvement policies such as increasing the reliance on renewable and clean energy and promoting electrical cars among others. These countries also should consider their international obligations of CO_2_ reduction when pursuing economic development.
‐ The international integration policies and strategies for CO_2_ emissions reduction should be emphasized and implement by countries from different income categories by establishing an effective collaboration and international cooperative mechanism. This can be the cornerstone in establishing a long‐range CO_2_ emissions reduction plan between all countries. To achieve this, high‐income countries must assess the low‐income countries in implementing these international pledges and agreements for environmental protection.
‐ It is important that high‐income countries should share emissions reduction technology as well as green technology innovation activities with low‐income countries. Deep collaboration and integration of technical endowment between countries from different income categories can achieve high‐efficiency CO_2_ reduction.
‐ In low‐income and lower‐middle‐income countries, policies on renewable energy, clean energy, and energy efficiency are important to reduce and displace fossil fuels. Initiating measures to decrease energy demand, increase investment in energy supply, and improve energy efficiency will not harm the economic development of the developing countries. Rather, these measures can allow them to meet their emission reduction goals without sacrificing economic growth. Amongst them, the GCC countries need to develop viable alternatives to oil and increase their funding for clean energy research and development.
‐ Finally, the issue of CO_2_ emissions extends beyond environmental considerations and encompasses various socio‐economic, equity, and developmental aspects. In order to achieve effective outcomes, it is imperative to employ comprehensive strategies that consider historical contexts, promote the development of environmentally sustainable innovations, and ensure that policies are firmly rooted in principles of equity and justice.

## Conflict of Interest

The authors declare no conflict of interest.

## Data Availability

The data that support the findings of this study are available from the corresponding author upon reasonable request.
